# Enhancing psychological safety in mental health services

**DOI:** 10.1186/s13033-021-00439-1

**Published:** 2021-04-14

**Authors:** D. F. Hunt, J. Bailey, B. R. Lennox, M. Crofts, C. Vincent

**Affiliations:** 1 Department of Experimental Psychology, Radcliffe Observatory, University of Oxford, Anna Watts Building, Woodstock Road, Oxford, OX2 6GG UK; 2 Oxford Health NHS Foundation Trust, Warneford Hospital, Warneford Lane, Headington, Oxford, OX3 7JX UK; 3 Department of Psychiatry, University of Oxford, Warneford Hospital, Warneford Lane, Headington, Oxford, OX3 7JX UK; 4 Oxford School of Nursing and Midwifery, Oxford Brookes University, Headington Campus, Oxford, OX3 0BP UK

## Abstract

**Background:**

Psychological safety—speaking up about ideas and concerns, free from interpersonal risk—are essential to the high-risk environment, such as healthcare settings. Psychologically safe working is particularly important in mental health where recovery-oriented approaches rely on collaborative efforts of interprofessional teams to make complex decisions. Much research focuses on antecedents and outcomes associated with psychological safety, but little focus on the practical steps for how to increase psychological safety across and at different levels of a healthcare organisation.

**Aims:**

We explore how a mental health organisation creates an organisation-wide plan for building the foundations of mental health and how to enhance psychological safety.

**Methods:**

This review encompasses strategies across psychological safety and organisational culture change to increase psychological safety at an individual, team and organisational level.

**Summary:**

We set out a comprehensive overview of the types of strategies and interventions for increasing the ethos of psychological safety and setting the foundations for delivering an organisation-wide programme on this topic. We also provide a list of key targeted areas in mental health that would maximally benefit from increasing psychological safety—both in clinical and non-clinical settings.

**Conclusions:**

Psychological safety is a crucial determinant of safe and effective patient care in mental health services. This paper provides the key steps and considerations, creating a large-scale programme in psychological safety with a focus on mental health and drawing from the current literature, providing concrete steps for how our current understanding of psychological safety into practice.

## Background

Psychological safety is the shared belief that it is safe to engage in interpersonal risk-taking in the workplace and is vital to team learning and performance, and facilitates willingness for workers to contribute towards a shared goal [1, 2]. Ideally, staff are free from the fear of being rejected for speaking up with suggestions and will be treated fairly and compassionately when discussing concerns, errors, or identifying problems. Not only to feel free from fear but also free from interpersonal, professional and social threats that could unfairly threaten their work status and future professional and occupational progression.

Psychological safety is particularly important in high-risk environments, such as healthcare, that rely on staff working in interprofessional and interdisciplinary environments where errors can result in significant harm or even death [3–6]. Despite the benefits of psychological safety, a culture of blame and fear is still prevalent in healthcare organisations, which is detrimental to patient safety, staff morale and organisational performance, leading to unreported errors and decreased patient safety [7–10]. This culture of blame and fear possibly compounded in countries that strictly adhere to hierarchical structures, where structure and control are paramount, with little to no opportunity for candid conversations across different organisation levels. Countries with market cultures may place competitiveness over the importance of discussing failures, creating a potentially toxic environment.

Psychological safety has an additional resonance and importance in mental health in empowering patients and families to voice their suggestions, concerns and anxieties. Many mental healthcare organisations adopt a recovery-oriented approach, focusing on empowering patients with the help of support structures (i.e., family and carers) to build on their strengths, make informed choices and play a central role in their health and other aspects of life [11].

This paper will discuss the benefits of creating a psychologically safe culture and then tackle the more difficult task of how psychological safety can be implemented organisation-wide. We first consider the challenges of cultural change of any kind, before addressing the particular challenge of enhancing psychological safety in mental health services. We set out a range of practical proposals to both support a broader organisational ethos of psychological safety and complementary initiatives which target settings that could benefit most from this approach. The design simultaneously considers building an ethos of psychological safety as well as targeted interventions that can have a measurable and impactful change.

## The challenges of cultural change

Organisational culture is the personality or spirit of an organisation. It is critical to the engagement and wellbeing of its workforce. More specifically, it is the collective manifestation of the shared beliefs, behaviours, thoughts, attitudes and norms that permeate throughout the workplace [12]. Schein describes culture as “the pattern of shared basic assumption—invented, discovered or developed by a given group” that new members receive as the “way we do things around here” [13, 14]. Importantly, this interpretation encompasses the observable socio-cognitive, interpersonal and symbolic manifestations of culture [15]. In this sense, organisational culture acts as the collective and is the potential driver of wider organisational innovation and change [16].

Despite the clear benefits of a positive organisational culture in healthcare, it has proved very difficult to achieve in practice and even more difficult to demonstrate. Recent systematic reviews investigating organisational cultural change on healthcare performance have not shown reliable results on its effectiveness [15, 17]. This is echoed in other research, with many attempts failing immediately or not sustaining over a long period [18]. Underlying these challenges is a longstanding debate whether it is possible to influence culture directly or whether it simply has to be taken into account, like the weather, when planning interventions and change [19].

Culture change in healthcare poses additional challenges. Healthcare needs and behaviours change over time to reflect the complex and diverse nature of patient needs, as well as increasing complexities in healthcare delivery. Typically, healthcare consists of different nested structures, some clinical and others non-clinical, with an executive core. Any team may deal with a different population, provide a different service or be part of several different services, and be placed within a particular location and form part of a particular site or be spread across multiple sites [20]. As well as team heterogeneity, healthcare organisations have multiple stakeholders’ interests and differing levels of interest that can present challenges to implementing consistent change. All of this has particular resonance and relevance when fostering a culture of psychological safety in a healthcare organisation.

On an international scale, the prevailing national culture will significantly influence whether cultural change is possible in any healthcare organisation in any given country. Factors such as individualistic vs collectivist ideologies, patriarchal vs matriarchal cultures, levels of tolerance of uncertainty will undoubtedly influence navigating cultural change in terms of what is achievable.

## Psychological safety in mental health

Creating a psychologically safe culture offers direct benefits to staff and the healthcare it provides, as well as making the foundations required for any future cultural changes. In healthcare, these benefits can be seen both in the day-to-day management and clinical practice and in providing the necessary foundations for longer-term improvement and innovation. In this section, we briefly set out areas which have particular relevance in mental health services.

### Speaking up and error management

Psychological safety plays a central role in detecting errors and near misses [1, 2, 21]. Speaking up is potentially particularly challenging in situations where there are intra-organisational (e.g., issues around patient safety and bed capacity) and inter-organisational (e.g., regulatory pressures from healthcare inspectorates) pressures.

The importance of speaking up is recognised internationally, with concerted efforts to remove barriers in healthcare organisations [22, 23]. Across countries and cultures, there are common barriers such as power and hierarchy, leadership influence, and concerns regarding the negative consequences of speaking up [23]. Most studies of psychological safety have been carried out in the United States and Europe, but the importance of speaking up to prevent errors has been recognised in diverse clinical settings across the world [22, 24, 25]. Patient safety teaching programmes and the World Health Organisation curriculum guide also recognise the critical role played by open communication within teams [26, 27].

In mental health, open and candid discussions are crucial as many clinical decisions are complex and ambiguous, and are a collection of subjective observations of a patient [28, 29]. Staff should not only be encouraged to discuss errors, but it should be an organisational cultural expectation. In return, staff should receive fair treatment and investigations into error will consider all contributing factors (e.g., staffing levels, patient acuity). Rather than error management just serving as an assurance tool for safe care, psychologically safe organisations use it as an opportunity to learn, to improve, and to calibrate expectations across its workforce.

The confidence to voice concerns is especially critical for patients, carers and families in mental health services. However, not all patients and loved ones feel able to discuss the difficulties that they have with their mental health issues or experiences of care. This is especially important as carers and families form an integral part of mental healthcare in the community. Psychologically safe organisations give patients, family, and carers the opportunities and space to have candid discussions and care pathways to be adapted to accommodate these discussions.

### Foundations of safety and quality improvement

Studies in other industries indicate a relationship between psychological safety and a capacity for rapid learning and innovation [30, 31]. Innovation and quality improvement (QI) rely on the workforce having the opportunity to feedback on problem areas that may require attention or that could be improved. Engaged staff who feel a collective responsibility provide intelligence on local need and effort in embedding change. Psychological safety is vital throughout all QI stages, from candid discussions when identifying problems, to taking controlled risks when experimenting and being free from fear of failure.

Psychological safety and its implications to QI are important in all countries. It is crucial in lower-income countries seeking to build and mature an effective healthcare workforce [32]. Both psychological safety and learning behaviours are key factors for the success of newly-formed QI teams in these settings [23, 32].

There has been a strong focus on QI in mental health, with many healthcare organisations shifting from away from assurance-based reporting. This approach has been reflected in healthcare inspectorates and regulators, such as the CQC’s evaluation of mental health in the UK, emphasising QI approaches [33].

Teams characterised by interpersonal trust and respect are more likely to engage in QI projects [21, 34, 35]. A psychologically safe organisation will understand the importance of learning from failure, and that as organisational changes are difficult, its workforce will understand the part they play in its success.

### Psychological safety and wellbeing

Promoting work-based wellbeing requires individuals to be able to recognise and report when they need help and are struggling with current work demands. Being able to admit that you need help can be viewed as a weakness with some being fearful that it may affect their reputation, job stability and future career prospects. However, not being able to speak up can lead to work-related stress, which can incubate this problem and lead to more significant health problems further down the line [36]. In mental health, speaking up about wellbeing may be incredibly difficult for staff as they may support people with similar challenges. Moreover, some staff may feel that speaking up about these issues will affect their perceived competence in carrying out their duties.

Confidence for healthcare staff to speak up is especially crucial during the COVID-19 pandemic, when many staff could be at risk of post-traumatic stress disorder or forms of moral injury, subsequently affecting their health and the care they provide (i.e., feelings of guilt in not being able to cope with current work conditions [37]).

## Principles of psychological safety

Studies of organisational change in general, and culture change in particular, suggest that several essential principles underlie any successful programme. To note, a recent systematic review discuss factors that enable psychological safety [4]. These principles focus on a whole system approach, considering behavioural change towards staff taking interpersonal risks in speaking up, leadership support to model and enable these changes and facilitating environmental and organisational changes. We summarise the main principles and success factors here, before turning to the practicalities of mapping and intervention.

### Psychological safety at every level

Psychological safety must be lived and experienced at every level of the organisation. This is clearly an ambitious and idealistic proposition but is vital as a principle even if it is hard to achieve in practice. Psychological safety will, however, be experienced and expressed in different ways according to the work context (Table 1).

**Table 1:** Implementing psychological safety at the individual, team, and organisation level

Level	Description
Individual	Feel that it is safe to report near misses or errors, suggestions for improvement; and Feel able to engage in discussions regarding the duties of their job and duties beyond their role for the benefit of patients and service users Feel empowered to discuss possible improvements and conduct controlled experimentation
Team	Emphasise compassionate and collaborative working Empowered to challenge disruptive or uncivil behaviours Learning and implementing suggestions for improvement and near misses and/or errors Invite innovation and experimentally testing suggestions to promote changes to processes for the future
Organisation	Executive-level leadership modelling psychological safety with strategic focus and investment Provide opportunities for individuals to engage in support networks and interprofessional working Promote management styles that are collaborative and compassionate Policies and procedures that emphasise fairness Enable and incentivise opportunities for improvement across the organisation

Executive leadership is essential for any large-scale change [38, 39]. Any organisation-wide programme requires engagement from the extended executive to simultaneously engage stakeholders from different directorates and core operations (i.e., HR, Governance). Executive buy-in is necessary at an early stage by discussing the research literature, options available, and developing an initial work plan with multiple streams. Furthermore, it increases the likelihood of obtaining an adequate level of investment at an early stage.

### Cultures and sub-cultures

Healthcare organisations are likely to be comprised of many subcultures [19]. The extent to which each subculture is psychologically safe will vary. Some teams may champion speaking up and open discussion, while others may be less psychologically safe. Staff may fear the risk of punishment or damage to their job security, engagement, and future job prospects. Some teams may be more willing to make changes that increase psychological safety. In contrast, some may feel resistant to change and hold on to current practice.

Creating a flexible psychological safety programme, refined to meet local need is crucial to the success of an organisation-wide programme. Indeed, teams vary in terms of their beliefs related to psychological safety. These can be influenced by variance in local manager styles and the known consequences in taking an interpersonal risk to speak up [2, 40]. Research underpins the importance of local leadership behaviours to enhance psychological safety; these behaviours include transformational leadership, leadership inclusiveness, managerial openness, trustworthiness and behavioural integrity [21, 41–44]. Furthermore, teams might vary in terms of the operational processes in place that facilitate psychological safety (e.g., meeting structures, content and frequency).

As well as recognising positive leadership styles, leadership values and behaviours should align with psychologically safe practice modelling throughout the organisation at an executive and local level. This approach requires a balance between not promoting direct and combative altercations within and between teams, but equally, not allowing unspoken issues and differences to fester and incubate into much larger problems in the future. As such, leaders at all levels must provide opportunities for subordinates to speak up, but equally to manage contributions positively and collaboratively. Moreover, leaders must also have the courage to temper or even thwart contributions that undermine psychologically safe practice. In other words, psychological safety is to promote collaborative and candid focused discussions and not a carte blanche approach, accepting any contributions. As well as the role of leaders in fostering psychological safety, it is also vital that they feel psychologically safe in their managerial duties and have HR practices that support them.

### Collaboration, co-design and co-production

Co-production demonstrates and utilises the value of experiential knowledge of staff, patients and their carers and families. This approach is a core practice that is commonly applied in health-related research [45, 46]. There are several connotations to the meaning of co-design/production in different contexts. For psychological safety, it is the collective responsibility in contributing to innovation and change that may lead to safer patient care. This includes contributing to suggestions for change, experimentation and providing feedback, and making efforts to implement changes into practice.

There are several reasons why this is important for the development of psychological safety interventions. First, it provides an opportunity for staff to participate in collaborating and co-designing interventions to apply their understanding of the local nuances to organisational plans, maximising chances of success [47]. Second, the experience of collaboration in itself can foster an experience of psychological safety and persuade staff of the sincerity of the intentions of executive leadership. Thirdly, co-design/production also increases the intrinsic motivation of staff and increases engagement in these changes and further promotes sustainability [48]. Finally, and related to the role of executive-level leadership support, co-design/production also places value in the involvement of staff, providing them with the opportunity to have the authority and feel empowered in supporting in increasing psychological safety.

## Understanding the current experience and practice of psychological safety

The first step in developing a programme is to assess the current state of psychological safety in the organisation, in terms of overall understanding and practice. Most organisations will also have other plans and initiatives already running, for instance, on staff well-being, which will overlap with the proposed programme on psychological safety. Mapping existing initiatives reduce the risk of duplicating work and, subsequently, maximises investment in changes related to improve psychological safety. This landscape mapping and scoping require a few key foundation steps (Box 1).

Box 1 First steps in psychological safety

**Table:** 

Assemble a small team to conduct the mapping exercise and will have access to key contacts within the organisation	
Establish a small steering group to guide the parameters of this mapping exercise, the ambitions and criteria for success	
Agree on an operational definition to identify what is a psychologically safe practice and what is not	
Establish a series of workshops, focus groups and interviews to explore current experience and perceptions of psychological safety with patients, families and staff	
Review relevant documents and procedures which may either support or detract from psychological safety	
Review training programmes, induction and other initiative both in terms of their ethos and content concerning psychological safety	
To establish a baseline measurement of psychological safety across the organisation

### Understanding the patient and family/carer experience

It is important to explore patient understanding of speaking up and their family/carer experience, who often form part delivering informal care. Unlike staff surveys, there is unlikely to be any large-scale surveys to formally capture the climate of psychological safety across all family members and carers involved in care. This is for several reasons. First, not all informal support is visible to the healthcare system (e.g., the sibling who supports their brother or sister when arriving home from school or work). Secondly, not all family/carers have access to the same methods of communication (e.g., email). Finally, this population is typically geographically disparate when compared to a healthcare workforce. The first step is to reach out to all active patients in the organisation to ask for participation. For particular groups, there may be gatekeepers that play an integral role in representing their population. Gatekeepers can include formal organisations such as large charities or local initiatives, or virtual social media support groups. Any focus groups or interviews should be at the convenience of patients and family/carers and should provide confidentiality. Messaging around these approaches is particularly important, clearly articulating that these experiences will inform mental healthcare delivery.

### Understanding the staff experience

Staff surveys (discussed below) will give a general picture of psychological safety across an organisation, but it is essential to complement this with a more nuanced understanding of staff views and experience of psychological safety. For example, a series of focus groups could be run with junior staff to explore their perceptions of speaking up. The experience of staff needs to be understood at all levels and sampled across all settings in the organisation. To fully engage with the workforce, it is essential that an accurate representation of perceptions of psychological safety, including barriers and opportunities. Those staff who feel trust in an organisation will be relatively easy to recruit, thus potentially biasing the findings. It is therefore critical to reach out to other individuals and groups who may be warier of speaking about their experiences. For example, introverted people, who may be less likely to speak up, but have equally valuable ideas than more assertive extroverts. It is therefore essential to gather feedback from those who do not typically speak up to gather the quiet power they bring in increasing psychological safety.

One way is to establish trusted gatekeepers who can serve to champion these initial discussions and facilitate in increasing confidence in speaking up, such as clinical leaders who may represent the protection of standards and quality. Engaging union representatives is a suitable method of reaching disenfranchised groups as well as provided reassurance of the confidential nature of such discussions. To further bolster confidentiality, focus groups can be held outside of regular working hours and at a neutral venue, so their participation remains confidential. Facilitators can be from an independent organisation or be a trusted person from the current organisation. For example, a chaplain from the organisation or union representatives are potentially ideal for facilitating these discussions. Telephone interviews also offer an alternative method of discussing this topic without the need to attend a venue and be recognised by others in the group.

### Review of core organisational policies and procedures

Organisational culture is primarily determined by the behaviour of people, particularly leaders, in that organisation. However, documents, procedures and symbols used by the organisation also express organisational culture. There are specific policies that would benefit from having a psychologically safe focus. For example, whistleblowing policies should embed psychologically safe practices to enable candid and fair dialogue between the whistle blower, those potentially implicated and the organisation. Encouraging staff to speak up is the first step, and organisational practices that support what happens after someone has spoken up is essential to sustaining these behaviours amongst the workforce. Policies relating to near misses should shift from being an assurance-based tool to encouraging and even rewarding staff that speak up, as well as promoting transparency, to show what learning and improvement are looped back into the organisation. Those policies enacting organisational change should take a similar approach, setting out an engagement approach to utilise local intelligence and gain buy-in from the workforce.

### Review induction and training programmes

Healthcare organisations provide different forms of education, both formal and informal, to all levels of the workforce. Many of the induction programmes include essential training on governance and information security, but other courses can consist of methods of care. For some roles (e.g., nurses, allied healthcare roles, and doctors), years of formal education has been completed as well as several placements. Local and organisation-wide induction training should focus on antecedents of psychological safety, such as team working, voice behaviours, and respectful listening [6]. Leadership programmes should have a strong focus on leadership behaviours such as inclusive, compassionate and collaborative leadership are integral to psychological safety [6, 31].

## Measuring psychological safety

Psychological safety is a complex multi-faceted concept and, subsequently, understanding the extent to which it has been a success and how this can be measured is a challenge. The most common form of measure for psychological safety is a team-level survey [1]. Others have adapted this survey to measure psychological safety at an individual- and organisation-level [42, 49].

Healthcare organisations typically send out staff surveys that are focused on different aspects of work experiences from their workforce. These surveys tend to cover categories that can serve as indicators for psychological safety (perceived managerial and organisational support, perceived compassion), so teams or services that may score low in these areas may also feel psychologically unsafe. For a major programme, however, it would be preferable to mount a specific survey of psychological safety at baseline and defined intervals as the programme unfolds. Burdening staff with additional surveys is of course, always a concern, but these are short and take only a few minutes to complete. Careful sampling strategies will also reduce the number of staff recruited to complete a new survey or adding questions to existing surveys. As well as producing longitudinal data, surveys can be useful in identifying groups of people who have scored low on psychological safety or who do not even feel able to complete a survey. These individuals and groups need particular support as executive leaders seek to gain trust across the whole organisation.

Objective measures of psychological safety will be beneficial for future research in this area. For example, observational frameworks relating to the verbal and non-verbal indicators of psychologically safe and unsafe practices might be particularly helpful in simulation interventions around speaking up and decision making. Once behaviours of psychological safety are agreed, behavioural markers provide ways to measure what is good or poor practice. Indeed, simulation-based education uses these frameworks to measure speaking up and assessing non-technical skills amongst medical teams [50, 51]. As such, observational frameworks behaviours provide an opportunity to measure behaviours reflective of psychological safety. In particular, to measure psychologically safe practice in some of the targeted interventions discussed below.

In the longer term, the fostering and enhancement of psychological safety should influence healthcare outcomes, such as improvements in patient safety and staff engagement. However, psychological safety is only one of many influences on such indices, and, therefore, it is challenging to measure a direct effect reliably. Assessing more immediate impacts, such as increased speaking up or reporting of near misses, maybe a more realistic earlier target. Furthermore, these targets can create a pathway to link the effects of psychological safety on ultimate outcomes such as safe patient care. Implementing a cultural change and increasing psychological safety will take a considerable amount of time, both in terms of a cultural shift with the existing workforce and inducting new staff. Staff surveys and evaluation of current practices over a long period offer an opportunity to realise the longer-term outcomes of a programme such as the one described.

## Enhancing psychological safety

Psychological safety is an intuitively straightforward and persuasive concept, though on reflection more complicated than it immediately appears. However, making meaningful, concrete steps to enhance psychological safety in an organisation is challenging for several reasons. First, psychological safety is multi-faceted, meaning that it requires a multi-faceted approach to change. Second, enhancing psychological safety requires a cultural shift, and any cultural initiative involves engagement and commitment from the majority of the workforce at all levels. Third, measuring psychological safety is especially challenging, in terms of how it influences ultimate outcomes such as patient safety, healthcare improvement and wellbeing. Finally, and most importantly, it is difficult to identify what concrete steps to take to enhance psychological safety, in what order and over what timescale. While there are many inspiring descriptions of organisations, who have embraced psychological safety, very little research provides any kind of defined set of steps or interventions. The journey of each organisation will be different, but it would be beneficial to define the essential components of a programme to enhance psychological safety.

Most psychological safety interventions aim to produce a broad change in attitudes, values and trust across the whole organisation. We refer to this generic approach as building an ethos of psychological safety. Targeted interventions, addressing settings and activities in which psychological safety is particularly critical, provide a complementary approach. Promoting a psychologically safe ethos should focus particularly on being a person-centred organisation, and a listening and learning organisation. A person-centred organisation will facilitate staff participating in creating an engaging workplace that focuses on safe patient care. A listening and learning organisation will make sure that they hear staff voices to discuss ideas for improvements, mistakes and errors, and contribute to failure-based learning.

The wider literature on psychological safety and organisational change suggest that there are a number of potentially useful means of exploring and influencing the experience of psychological safety. In Box 2 we set out the target actions for increasing an ethos of psychological safety, and in Table 2 we set out methods for implementing these actions, based on literature relating to psychological safety interventions and organisational change interventions [4, 15, 17, 52, 53].

**Table 2:** Techniques and practices to enable cultural and organisational change

Intervention	Actions	Evidence
Organisational and leadership messaging	Create a communication strategy using multiple approaches to reach the whole workforce and the wider community. Strategies include sending letters and factsheets, and in-person roadshows	Letter and fact sheet campaign [54]. Leadership videos [55]
Developing an organisational charter	Code of conducts can provide a framework for behavioural expectations. Codes of conduct offer the opportunity to embed behaviours taught in workshops and educational sessions (see below) into current practice	Team charter to empower teams [56]. Code of conduct [57]
Mental health ethics committee	Open and accessible ethics committees provide objective and supportive scrutiny, particularly around explore complex dilemmas and experimenting with new approaches to patient care (i.e., service change and quality improvement)	Ethics committees and consultations [58, 59]
Dialogue meetings	Create a series of dialogue meetings to open up questions that are frequently either unanswered or unanswerable to generate open discussion on difficult topics in mental health	Opportunities to discuss complex dilemmas [60, 61]
Schwartz rounds	Provide interdisciplinary meetings for groups to discuss the emotional and social aspects of care with the purpose of providing safer patient care. It is led by a multidisciplinary panel who open with their experiences around a particular theme. Schwartz rounds are complementary to dialogue meetings, focusing on share experience and compassion instead of unearthing the thought processes regarding difficult situations	Schwartz rounds in mental health and community care [62]
Staff engagement and action research groups	Town halls offer an opportunity to bring together representatives across the organisation to discuss psychological safety. Action research groups, allowing clinicians to act as researchers, calibrate approaches and share best practice	Staff engagement [63, 64]. Action Research Groups [64, 65]
Patient participatory councils	These groups provide an opportunity to bring professionals, managers and clinical and non-clinical staff together to ensure healthcare is patient-oriented and maximise patient involvement and choice	Patient engagement and participatory action research group [66]
Skills workshops	Workshops and train the trainer workshops offer opportunities to bolster skills and embed this into practice by training champions	Train the trainer workshops [57]. Skills workshop [67, 68]
Simulation and role play	Utilising the role of simulated or unstructured role-play to explore complex scenarios in mental health in safe no-risk environments	Simulation [51]. Role-play [69]
Video presentations and case studies	This approach provides teams with the time to self-reflect and reflects on everyday events that are complex and generally made under pressure	Video dramatisation of medical events [70]. Vignettes [71]

Box 2 Areas of action to increase a culture of psychological safety

**Table:** 

1. Select and engage a core group of key influencers in the organisation to lay out a strategic plan with the core columns of psychologically safe practice. Pillars for ultimate outcomes and cross-cutting themes for the requirements to achieve these outcomes. A visual representation of this is below:	

2. Support and commitment to psychologically safe practice from the organisation to their workforce. This approach includes communicating this commitment to the workforce and the wider community, solidifying the organisational commitment to psychological safety to the proposed strategic plan	
3. Leadership messaging to model psychological safety and focus on the following topics: (a) Discussing the importance of reporting failures and benefits from focusing on quality improvement. Providing empirical substantiation to create an impetus for change (b) Discuss the collective responsibility of staff to speak up when delivering safe patient care and areas that can be improved, including plans to make the process of reporting fair and straightforward. This can also include actively congratulating and even rewarding these actions, where appropriate (c) Discuss personal experiences of occupational failure and what learned lessons from these experiences. This will serve to make failure an acceptable and model that it is acceptable and is part of occupational development (d) Discuss previous difficulties in speaking up to senior colleagues, lessons learned and the importance of speaking up and shared decision-making within and between teams and professions	
4. Create a code of conduct to set expectations for how people should act with each other and instil the values of psychological safety, including open and candid discussions, balanced with compassion and fairness: respectful listening and collaborative debate, all with the focus on providing safe and optimum patient care	
5. Forums and structured discussions for intra- and inter-professional groups to discuss challenges and opportunities in mental health practice. These could provide opportunities to: (a) Create a series of groups that target particular professions, services or problems related to psychological safety. Each group should include an appropriate sponsor who will commit to championing actions arising from these discussions (b) Discuss the collective responsibility of staff to speak up when delivering safe patient care and areas that can be improved. Crucially, these discussions should directly feedback into the organisation and take an action research approach to create improvements from these discussions (c) Provide opportunities to discuss complex decision-making and inform action research that involves staff in co-designing interventions to enhance behaviours relating to psychologically safe practice	
6. Provide training that focuses on psychologically safe behaviours and practice. These include speaking up and voice behaviours, autonomy, respectful listening, collaborative working, advocacy enquiry and collaborative debate	
7. Create medical, educational interventions that allow teams to practice psychologically safe practice in clinical and non-clinical situations. These can include simulating high-pressured situations such as aggressive and violent patients

**Targeted interventions**

Psychological safety culture change requires a broad approach to instil the ethos through all layers of the organisation, creating a consistent message and support for this approach. However, alongside this, targeted interventions provide an opportunity to create test beds where psychological safety is vitally important. There are ‘pinch points’ when it is particularly important for patient, family and staff engagement and the delivery of care. Psychological safety is particularly important, when coercive measures have to be used to protect the patient from harming themselves or other people. The trauma and distress that such measures may provoke can be eased by open and compassionate communication at the time and by careful debriefing and explanation afterwards when the crisis has passed. Whilst debriefs tend to be a statutory requirement, they provide an opportunity for increased staff reflexivity, empower patients to contribute to their care (e.g., discuss future ways of using alternative options to restrictive practice) and enhance patient outcomes [72].

In the broadest sense, targeted interventions fall into two categories. The first is structured situations such as meetings that provide an opportunity to speak up about areas of concern or possible improvement. The second is where decisions are made in situ and take place on an ad hoc basis and require a group discussion. When a decision is complex, and there is no obviously correct course of action, it is particularly critical that patients, families and staff all feel able to speak openly and contribute to the decision-making process. Table 3 provides some key target areas for psychological safety, both in a clinical and non-clinical setting.

**Table 3:** Key target areas for psychological safety

Situation	Description	Relevance of psychological safety	Example interventions
Patient admissions to a psychiatric hospital	Decisions to admit patients depend on system demands (both intra- and inter-organisational), patient presentation and capacity to consent to admission, country-specific legal frameworks and ward resources to care for the patient compassionately and safely. A patient-centred focus can leave nurses feeling that they must always admit patients, irrespective of resource levels, and do not have the opportunity to decline further admissions when they assess safety to be compromised	Psychologically safe teams can discuss decisions that could be viewed as incorrect, subversive or unhelpful and do not align with the intra- and inter- organisational demands, and individual desire to aid all patients. Not only will nurses be empowered to make the difficult decision to decline admission, but leadership will support these decisions	Daily or shift based patient flow meetings, and dialogue meetings to provide staff with the opportunity to openly discuss internal and external demand for capacity in their clinical areas, their concern for impact on standards of care, their compassion for the person needing admission, their safety-based decisions and calibrate these approaches Leadership training focused on the importance of making patient safety-focused decisions regarding admissions and empowering teams to decline further admissions when it is unsafe for patients and staff to increase care demands on the ward team
Involuntary admission to a psychiatric hospital	Most countries provide statutory powers to admit the most vulnerable into psychiatric inpatient services, and appropriate treatment that may arise from this decision. Involuntary inpatients can feel disempowered and this can potentially influence their recovery	Psychologically safe teams will recognise the importance of seeking shared decision-making with involuntary inpatients. These discussions should include discussing patient options, their preferences, and openly discussing their preferences set against what is possible and safe	Develop and implement a new structured induction plan (or refining an existing one) for all involuntary admissions that focus on patient choice Communication training that specifically focuses on negotiating with patients and factors in patient preference against what is feasible for their safety
Decisions to use pro-active intervention	In acute inpatient settings, these situations are the precursor to restrictive practice, such as discussing and debriefing patients, providing an opportunity to support them in de-escalating a situation In community settings, these situations may include introducing a pharmaceutical or therapeutic intervention to help stabilise a patient and avoid admission to a psychiatric hospital	Psychologically safe teams in acute inpatient care (particularly with nursing and practitioner teams) will actively seek to discuss and take action to de-escalate volatile situations, providing support in these actions and not avoiding them In community settings, psychologically safe teams will involve all relevant professionals, informal support and the patient in openly discussing intervention options available to provide patient-centred care	Dialogue meetings provide opportunities for teams to discuss these difficult decisions and their thought processes, relating to previous experiences Schwartz rounds provide an opportunity for staff to discuss the emotional components of complex decision-making, particularly balancing patient safety and privacy Simulations and role-play provide an opportunity for teams to enhance psychological safety behaviours such as team decision-making, diverse thinking, teamwork and speaking up to deliver patient-focused care. Simulations should also include shared decision-makers with key stakeholders beyond the team (e.g., informal support, non-statutory organisations). Most importantly, involving the patient in shared decision-making, both in terms of keeping them informed, providing patient choice and negotiating patient preference with patient safety considerations Debriefing teams to explicitly discuss thought processes and factors relating to positive risk-taking behaviour. Alongside this, modelling thoughtful risk-taking behaviour from leaders, including a protocol to consider all factors
Decisions to use restrictive practice in acute inpatient settings	Across acute inpatient settings, decisions on the use of restrictive practice (e.g., enhanced observations, seclusion, restraint, and sedation) are often time-pressured and involved complex decision-making, making a trade-off between patient safety and privacy. As such, these decisions require input from all key decision-makers and influencers to make the most informed choice	Teams that are high in psychological safety will be able to speak up and voice their considerations and possibly their concerns on the use of restrictive practice, including challenging the decisions made by others in a collaborative way. As well as speaking up, psychological safety teams will include all members participating in the decision-making process, and ensure full patient involvement	
Reducing restrictive practice in acute inpatient settings	Related to the decision to use restrictive practice is to de-escalate or end restrictive practice with a patient. This involves testing whether a patient is able to manage without the imposed restraints. It requires positive risk-taking behaviour to increase patient privacy but not at the expense of the safety of the patient and others	Psychologically safe teams support and collaboratively challenge each other when it comes to patient-focused and positive risk-taking (i.e., experimenting whether a patient can manage without an environmental restraint). Utilising whole team intelligence, full patient involvement (both in terms of informing the patient and discussing the available options) and knowledge sharing enhances the decision-making, mitigates foreseen risks and encourages taking positive risks that benefit the patient	
Authorising leave or time away from the ward in acute inpatient settings	As part of a recovery-oriented approach, many patients have planned leave or time away from the ward in the context of the legal framework under which they were admitted to hospital. This positive risk-taking requires ward teams to balance the often complex dynamic between patient autonomy and organisational/societal paternalism		
Post-incident debrief/discussion for the use of restrictive practice	Across many countries, debriefing is a mandatory (and legally required) to analyse and discuss all circumstances leading to the decision to use restrictive practice	A psychologically safe team can have candid discussions regarding the use of restraint, even when this practice was not optimal the optimal decision for the patient (e.g., low staffing levels creating a need to use more restrictive practice). These teams should also be able to collaboratively discuss any disagreements regarding decisions made, in retrospect Teams will also seek patient involvement and family/carer involvement (where applicable) in individual cases, to reflect on the decision made, discuss the reasons why they were made, and actively seek a collaboration to explore alternative options and strategies for avoiding using restrictive practice, where possible	Training provided to teams that support speaking up candidly and communication styles to reflect collaborative rather than combative debate Organisational policies in place to fairly audit non-optimal decisions regarding restrictive practice, fairly and holistically Train and simulate shared decision-making approaches, inclusive of patients in these discussions
Planned discharge from a psychiatric hospital	The transition from inpatient to the community can be a difficult inpatients, particularly for patients with long lengths of stay	A psychologically safe approach to the transition in care between inpatient and community settings will involve the patient in discussing the plan, openly and actively listening to any concerns the patient may have, and to ensure continuity of care between inpatient and community teams Involving all relevant statutory and non-statutory organisations, and informal support in the planned discharge	Dialogue meetings and reflective sessions to understand the barriers and discuss ways of ensuring continuity of care for this transition period Mapping and refining the transition aspect of a care pathway to ensure full patient involvement and involvement of relevant services and informal support
Decisions regarding particularly vulnerable groups	Decisions regarding the care of vulnerable groups such as older people and young people require collaboration between clinician’s informal support and the views of the person themselves. In particular, the process of making decisions regarding the care provided by carers, partners, family and friends vs what professional assessment determines to be the best approach This should also include other statutory and non-statutory organisations that are involved in supporting the patient As such, patient-centred care often crosses professional and organisational boundaries, requiring open and candid communication for inter-professional working, and shared decision-making that involve patients and their informal support network	Safe patient-focused care relies on the continuity of care and collaboration between professional services and other statutory and non-statutory organisations (e.g., charity-sector organisations involved in supporting a patient); and informal support provided by carers, friends and family. Psychological safe teams can engage in supportive and candid discussions between formal and informal care to agree to care packages that are always in the interests of the patient	Engagement strategies and training that bring together formal and informal support to integrate and create continuity of care—providing opportunities for people to speak up about parameters, opportunities and challenges to providing care
Clinical handover	The definition of clinical handovers is the transfer of clinical responsibility and accountability of some or all aspects of care for a patient or group of patients to another person. Functional handovers underpin consistent and continuity of care. In the case of an inpatient setting, this handover will typically be at the end of one shift pattern to the beginning of another. Handovers should include enough information to plan for the next shift compassionately and safely	As such, teams that are high in psychological safety will be able to have candid and pro-active discussions that could aid the effective care of particular patients. For example, speaking up about some of the behavioural difficulties with one patient may provide insight that helps to avoid an aggressive situation later that day	Create a research action team with key decision-makers (clinical and non-clinical staff) across the Trust to map how handover process and discuss restructuring it with a focus on psychological safety
Informal disputes between staff members	Providing opportunities and structure to handling disputes between staff members is essential to resolving disputes before they reach formal grievance procedures	Psychologically safe teams provide an opportunity for staff members to have candid conversations about professional conflicts and seek opportunities to resolve such issues successfully. In particular, a focus on understanding each other’s view and recognising commonalities in occupational practice. Where possible, staff should take opportunities to take ownership of the issue and seek to resolve it in an appositive manner At an organisational level, there should be structure and opportunities to facilitate staff resolving such issues at the early stages	Specific training provided to staff members and, in particular, to leaders to help mediate and resolve disputes and conflicts. Training should also focus on respectful listening and divergent thinking Organisations may seek to provide mediation from internally-appointed individuals or external services to facilitate pro-actively handling disputes
Workplace bullying and harassment	Individuals who misuse their authority to gain personal and political power, and ultimately undermine a psychologically safe culture At a group level, informal peer support networks are beneficial in a workplace setting, they can also serve as cliques, creating favouritism for those within the group and exclusion for those outside of the group	Psychologically safe teams create professional boundaries, understanding the difference between their professional and personal lives. Irrespective of informal relationships, colleagues still foster a relationship of candid discussion that is equal across all colleagues. When witnessing bullying and harassment, they are openly willing to challenge this behaviour and follow through with any action arising from the incident	Creating a code of conduct in which team expectations are agreed upon, laid out and repeatedly reinforced Policies that protect and support those who are implicated in bullying and harassment incidents, both for the person who reports the incident and the implicated staff Leadership training with a specific focus on handling bullying and harassment, from proactive strategies to promote candid but fair conversations, to handling grievances arising from this any reported incidents
Grievance procedures	All organisations have formal positions in place to handle grievances, whether they are through internal HR processes or whether they involve independent union organisations	Psychologically safe teams will be able to have candid conversations about any dispute or differences in personality or workplace differences that, in most cases, can be resolved before reaching formal stages	Providing independent and informal routes specifically designed to work towards resolving grievances collaboratively. Creating local charters with expectations for staff to openly, candidly and respectively discussing grievances before they escalate to formal stages
Professional Development Review (PDR)	Professional development reviews (PDR) provide opportunities for both the staff and the managers to reflect on performance from the previous year as well as plans for the next year. It provides opportunities to engage in short-term planning for role fulfilment and long-term career aspirations and how the organisation may play a part in this	Those who are psychologically safe will be free to engage in candid conversations regarding where they want to be in their position and will facilitate their engagement. The PDR should primarily seek to serve and develop the individual, and for organisational performance viewed as a secondary focus. It can provide opportunities for staff to discuss how the role fits with their current career aspirations and how this fits with the current role and what the organisation needs	Review of current PDR across the organisation and interviews to discuss whether staff the plans reflect their true aspirations, goals and plans
Return to work interviews	Return to work interviews are short, informal meetings held with an employee on their return to work after an absence. As well as discussing details around the absence and the planned return to work, it provides an opportunity to explore any work-placed contributions	Psychological safe individuals will be able to speak candidly around any work-related stress that contributed to their absence. Receptive managers will be able to respond to these concerns and, where possible, provide a return to work plan that takes into account the contributions to absence	Specific training that focuses on psychologically safe behaviours enabling collaborative discussion about the current absence, broader influences, and candidly discussing next steps
Exit interviews	Exit interviews create an invaluable opportunity to find out whether any of the reasons staff leave is attributable to the organisation. On a population level, they also offer opportunities to recognise trends and areas that may be improved to increase staff retention and engagement	Even when leaving, employees may feel like they are unable to speak freely about the reasons they are leaving—fear of not receiving a good reference further influences whether people speak up. Individuals high in psychological safety are likely to speak more frankly about their experience and can offer critical insights for an organisation. Like PDRs, an exit interview should seek to serve the individual, first and foremost, and to provide open opportunities for organisational learning	Develop and implement a process to thematically analyse patterns for people leaving and how this is fed back into the organisation—alongside this, messaging that feedback received is valuable and actioned

In the targeted interventions discussed, three common themes emerge in mental healthcare practice. The first is the importance of empowering patients by keeping them informed at every stage of their care and providing patient choice wherever possible. The second is the importance respecting and encouraging the contributions of all healthcare staff that help deliver patient-centered and recovery-oriented care. The third is understanding that in difficult situations, such as disputes or grievance procedures, there should be an expectation of candid, open, and fair conversations that are collaborative in nature and not combative, focusing on individual development and team development.

## Conclusion

Psychologically safe practice is essential in mental health to innovative practice and safe patient care, provided by a healthy and engaged workforce. Despite psychological safety, being an intuitive concept to understand, operationalising it at scale is particularly challenging. It has a particular resonance in mental health for two reasons. First, many mental health organisations focus on recovery-oriented practice which requires substantial patient and family involvement. Second, decision making in mental health is often complex and ambiguous, based on subjective observations that require whole team input. As such, assuring all parties feel free to speak up and have maximum involvement is vital to safe and optimum mental health patient care.

This overview and proposed plan for enhancing psychological safety largely focuses on the UK mental healthcare system and may not be applicable to healthcare settings in different countries. Indeed, healthcare organisations will differ in terms of their structures, levels of investment and prevailing cultures, meaning that not all aspects of this plan are applicable in different countries or cultures. Despite these differences, many of the challenges and suggested approaches will translate on across countries and cultures. For example, the importance of speaking up about errors or ideas for improvements, the barriers are common across different countries and cultures.

As well as staff engagement, establishing a council of patients and actively encouraging family/carer participation is possible in all settings, even if this is more challenging in some cultures. As such, whilst the plan itself may not be applicable to different and more disparate healthcare organisations, many of the suggestions can be applied individually or tailored to be applicable to different settings.

Future studies may explore methods for implementing psychological safety in non-traditional organisational research settings, and factor in the recognised differences in culture and existing structures. For example, one might envisage healthcare organisations may differ in societies strong in collectivist vs individualistic ideologies.

In this paper, we discuss how to create the foundations of psychological safety and the importance of preparatory stages from a structural and cultural perspective. Following this, we propose a practical guide that split psychological safety into two categories: building an ethos across an organisation and target areas, including some specific to mental health. This paper seeks to provide two advancements. First, it can serve as a ‘blueprint’ for healthcare organisations to approach enhancing psychological safety in a meaningful way. Second, it provides suggestions for research to be advanced in psychological safety, with a particular focus on what possible routes for development. This paper, therefore, serves as a primer for approaching psychological safety and forms a bedrock for further development on this topic, from a mental health perspective.

## Data Availability

Not applicable.
